# Sphingolipid Pathway as a Source of Vulnerability in IDH1*^mut^* Glioma

**DOI:** 10.3390/cancers12102910

**Published:** 2020-10-10

**Authors:** Tyrone Dowdy, Lumin Zhang, Orieta Celiku, Sriya Movva, Adrian Lita, Victor Ruiz-Rodado, Mark R. Gilbert, Mioara Larion

**Affiliations:** 1 Neuro-Oncology Branch, National Cancer Institute, National Institutes of Health, Bethesda, MD 20814, USA; tyrone.dowdy@nih.gov (T.D.); lumin.zhang@nih.gov (L.Z.); orieta.celiku@nih.gov (O.C.); adrian.lita@nih.gov (A.L.); victor.ruizrodado@nih.gov (V.R.-R.); gilbert.mark@nih.gov (M.R.G.); 2 George Washington School of Medicine and Health Sciences, Washington, DC 20052, USA; sriyamovva@gwu.edu

**Keywords:** N,N-dimethylsphingosine, sphingosine, sphinganine, IDH*^mut^* gliomas, sphingolipid metabolism

## Abstract

**Simple Summary:**

The presence of the IDH mutation in glioma raises the possibility that these CNS malignancies could be targeted with metabolic-based therapeutics, however, the exploration of the role that regulatory lipids, such as sphingolipids serve within the IDH1^mut^ gliomas remains limited. Our study aimed to identify vulnerabilities within the sphingolipid metabolism that could be exploited therapeutically. We revealed elevation in certain lipids produced along the sphingolipid pathway for IDH1 mutated glioma cells and upregulation of the corresponding enzymes. We demonstrated that inhibiting sphingosine kinase which exacerbating the imbalance between sphingosine and sphingosine 1-phosphate leads to cell death, specifically for IDH1*^mut^* gliomas. These findings present potential to translate into targets for the development of metabolic therapies that may promote improved prognosis for patients diagnosed with IDH1*^mut^* gliomas.

**Abstract:**

In addition to providing integrity to cellular structure, the various classes of lipids participate in a multitude of functions including secondary messengers, receptor stimulation, lymphocyte trafficking, inflammation, angiogenesis, cell migration, proliferation, necrosis and apoptosis, thus highlighting the importance of understanding their role in the tumor phenotype. In the context of IDH1*^mut^* glioma, investigations focused on metabolic alterations involving lipidomics’ present potential to uncover novel vulnerabilities. Herein, a detailed lipidomic analysis of the sphingolipid metabolism was conducted in patient-derived IDH1*^mut^* glioma cell lines, as well as model systems, with the of identifying points of metabolic vulnerability. We probed the effect of decreasing D-2HG levels on the sphingolipid pathway, by treating these cell lines with an IDH1*^mut^* inhibitor, AGI5198. The results revealed that N,N-dimethylsphingosine (NDMS), sphingosine C17 and sphinganine C18 were significantly downregulated, while sphingosine-1-phosphate (S1P) was significantly upregulated in glioma cultures following suppression of IDH1*^mut^* activity. We exploited the pathway using a small-scale, rational drug screen and identified a combination that was lethal to IDH*^mut^* cells. Our work revealed that further addition of N,N-dimethylsphingosine in combination with sphingosine C17 triggered a dose-dependent biostatic and apoptotic response in a panel of IDH1*^mut^* glioma cell lines specifically, while it had little effect on the IDH*^WT^* cells probed here. To our knowledge, this is the first study that shows how altering the sphingolipid pathway in IDH1*^mut^* gliomas elucidates susceptibility that can arrest proliferation and initiate subsequent cellular death.

## 1. Introduction

Gliomas are devastating brain tumors with no known curable treatment. Despite decades of progress in this area, the overall survival prognoses for patients diagnosed with these tumors have not improved drastically. Gain-of-function mutations in isocitrate dehydrogenase I (IDH*^mut^*) are very common events in lower grade gliomas and secondary glioblastomas, causing overproduction of D-2-hydroxyglutarate at millimolar concentrations [1,2,3]. Although, overall patients with low-grade IDH*^mut^* gliomas are known to present increased survival compared to both low- and high-grade glioblastomas, this survival could be improved with additional molecular therapies [4,5,6]. The presence of a metabolic alteration in these gliomas raises the possibility of implementing metabolic-based therapeutics; however, a comprehensive understanding of metabolic vulnerabilities within the IDH1*^mut^* gliomas is limited. Since IDH*^WT^* is a central enzyme of the tricarboxylic acid (TCA) cycle, many investigations have explored targeting of central carbon metabolism in these tumors. As a result, several targeting approaches have been proposed within related pathways which include targeting glutaminase [2,7], nicotinamide adenine dinucleotide (NAD) metabolism [8], exploiting the increased reactive oxygen species (ROS) intrinsically present in IDH1*^mut^* gliomas [9,10], as well as the direct inhibition of IDH1*^R132H^* mutant activity [10]. While the central carbon metabolism has been studied extensively in cancer, and specifically in gliomas, the lipidomic analysis is still sparse. Very few investigations are focused on lipids while even fewer are focused on sphingolipids within the IDH1*^mut^* gliomas, despite their important roles in oncogenic signaling [11].

Ceramide and sphingosine 1-phosphate (S1P) are amongst the most studied sphingolipids. Ceramide and sphingosine maintain a negative correlation with S1P via the sphingolipid-rheostat balance, in which the upregulation of ceramide is directly correlated to a concomitant downregulation of S1P and vice versa [12]. The fate and accumulation of sphingosine is a major driver in the sphingolipid rheostat balance, which directly modulates cell fate [11,13]. Moreover, sphingosine has been implicated in anti-proliferative signaling in a manner that resembles ceramide-induced apoptosis in certain cancers [12,14]. Sphingosine can inhibit protein kinase C and phosphatidic acid phosphohydrolase, whose activities promote the oncoprotective, anti-apoptotic response in certain cancers [15,16]. Moreover, sphingosine stimulates phospholipase C-protein kinase C-mediated activation of phospholipase D, which is attributed to inducing apoptosis in certain cancer cells [17].

While certain sphingolipids (e.g., analogs of ceramide, sphingosine and sphinganine) can serve as bioeffectors that disrupt pro-survival cellular signaling pathways, S1P has been classified as an oncopotent biomolecule which mediates cell signaling as an intracellular second messenger and a ligand of G protein-coupled receptors (GPCR) [18,19]. S1P utilizes multiple targets to stimulate complex cellular processes involving chemotactic motility, migration, differentiation, proliferation, apoptotic survival response, capillary permeability, differentiation and angiogenesis, which are key hallmarks of cancers [18]. Noteworthy, endogenous analogs of S1P (phytosphingosine 1-phosphate, dihydrosphingosine 1-phosphate and ceramide 1-phosphate) are also bioeffectors that modulate the pro-survival response in tumors, especially [19,20,21]. Despite known roles of ceramide, sphingosine and S1P in multiple cancer types, their contribution to IDH1^mut^ gliomas remains unknown. Given their historical, vital roles in cell signaling and regulation, we hypothesized that manipulating the dynamic relationship between ceramide, sphingosine and S1P would reveal vulnerabilities and potentially translate into therapeutic targets to combat proliferation in IDH1*^mut^* gliomas.

Our interest in the sphingolipid pathway originated from their well-documented signaling roles described above as well as our recent findings related to this pathway. Using organelle lipidomics and Raman spectroscopy, we showed that phospholipids and sphingomyelin are significantly increased in the organelles of IDH1*^mut^* glioma compared with their IDH*^WT^* counterpart [22]. These findings pointed us to investigate the sphingolipid pathway in greater detail. Therefore, herein, we undertook a lipidomic comparison of patient-derived IDH1*^mut^* neurospheres untreated and treated with *IDH1^mut^* inhibitor, AGI5198, in order to identify common alterations in this pathway. We found that sphingosine 1-phosphate and phyto-S1P were significantly increased in the presence of AGI-5198, the inhibitor known to suppress D-2HG formation, while N,N-dimethyl sphingosine (NDMS), sphingosine-C18, sphingosine-C17 and sphinganine-C17 were decreased. We validated our findings in a model system where U251 glioblastoma cells were engineered to overexpress either IDH*^wt^* or IDH1*^R132H^* genes, from which we measured sphingolipids located in the endoplasmic reticulum (ER), a site of their biosynthesis. We then designed a rational drug screen targeting this pathway and identified that combining NDMS with sphingosine-C17 increased the efficiency of arresting cellular proliferation and triggering cell death. Our findings indicate that targeting the sphingolipid metabolism may present a promising strategy to improve survival for patients diagnosed with IDH1*^mut^* gliomas.

## 2. Results

### 2.1. Sphingolipid Pathway Is Commonly Dysregulated in Astrocytomas and Oligodendroglioma Expressing IDH1-R132H Mutation

**Figure 1:**
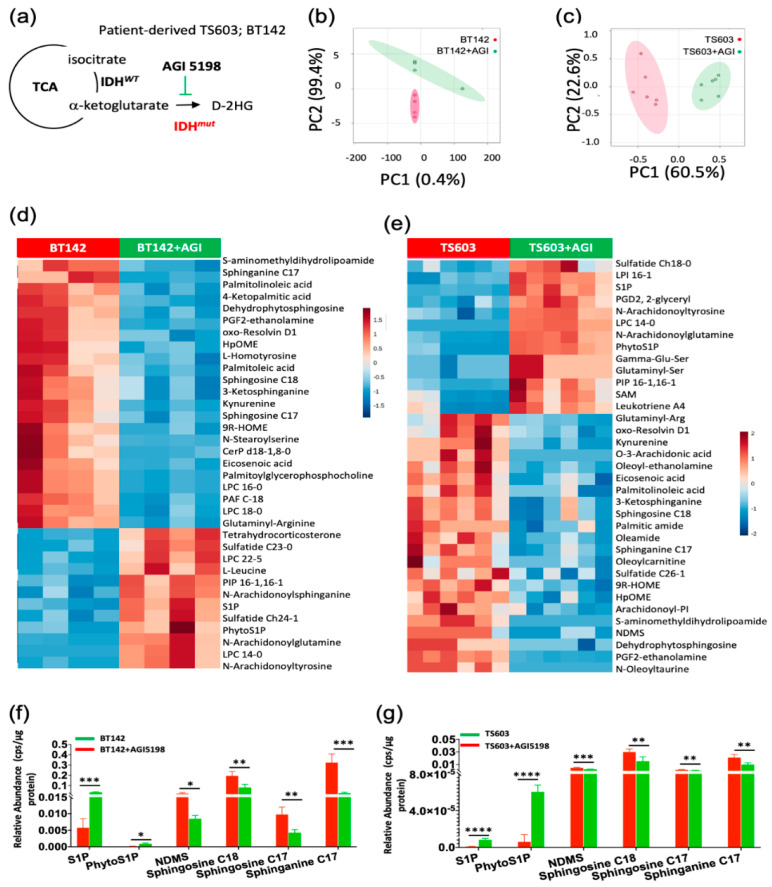
Discovery of sphingolipids pathway altered in response to suppression of D-2HG formation of patient-derived IDH1*^mut^* cell lines (**a**). Schematic representation of the reaction catalyzed by IDH1*^mut^* prevalent in glioma and the strategy utilized in this manuscript. BT142 and TS603 are cell lines derived from either astrocytoma or oligodendroglioma patients respectively and were treated with 50 µM AGI5198 to probe common metabolic alterations associated to inhibition of IDH1*^mut^* activity in both cell lines. (**b**) 2D PCA plot for the untreated BT142 (red) and AGI5198-treated cells (green) (*n* = 4). (**c**) 2D PCA plot for the untreated TS603 spheroids (*n* = 6) (red) and AGI5198-treated (green) TS603 cells (*n* = 6). (**d**,**e**), heatmaps displaying differentially regulated lipids in IDH1*^mut^* control (red) versus AGI 5198-treated cells (green). Heatmaps were generated using MetaboAnalyst 4.0 [23]. (**f**,**g**), bar plots of sphingolipids that displayed the same trend upon AGI-5198 treatment (green) in both cell lines. Statistical analysis (t-test) was conducted assuming equal variance, *p*-values are represented as follows: **** *p* < 0.00005, *** *p* < 0.0005; ** *p* < 0.005; * *p* < 0.05; ns, not significant.

To probe the alterations in the sphingolipid pathway, in this study, we utilized patient-derived cell lines which endogenously express the R132H variant of IDH1. While these models are rare and very valuable, their genetic heterogeneity makes the metabolomic results difficult to interpret. To overcome this limitation, we conducted a 72 h treatment for mutant IDH1-R132H BT142 (astrocytoma) and TS603 (oligodendroglioma) cell lines with AGI5198, which specifically inhibits IDH1*^R132H^* enzyme activity disrupting D-2HG formation (Figure 1a). This in vitro approach facilitated the probing of differentially altered lipids when production of endogenous levels of D-2HG were suppressed. We then conducted a multivariate analysis using our LC/MS lipidomic platform optimized for the detection of polar lipids (primarily, sphingolipids). Principal component analysis (PCA) plots corresponding to both cell lines showed a high degree of separation between untreated and treated groups (Figure 1b,c). Following metabolite assignments, we identified the top 50 most altered species that were mainly composed of sphingolipids in both genetically distinct cell lines. The relative abundance analysis in the heat maps provides evidence of a functional relationship between the D-2HG-driven metabolic reprogramming and sphingolipid pathway. Regardless of the genetic context, we noticed a conserved trend in the sphingolipid pathway (Figure 1d,e, Tables S1 and S2). Both cell lines showed significant elevation in S1P and phyto-S1P when D-2HG concentration was suppressed by AGI5198 (Figure 1f,g).

On the other hand, N,N-dimethylsphingosine (NDMS), sphingosine-C18, sphingosine-C17 and sphinganine-C17 showed a significant decrease in their levels upon addition of IDH1*^mut^* inhibitor, AGI5198. These results suggest a direct correlation between D-2HG and global sphingosine/sphinganine levels along with an inverse correlation to S1P levels.

### 2.2. Probing the Sphingolipid Pathway

**Figure 2:**
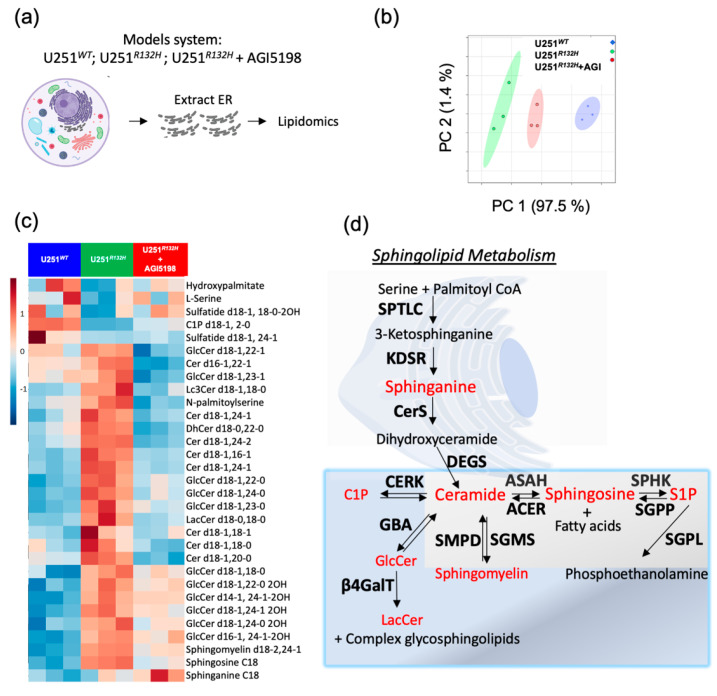
Sphingolipids pathway is upregulated in the endoplasmic reticulum of U251 cells overexpressing mutant IDH1-R132H variant and it is downregulated in response to suppression of D-2HG formation in this model cell line. (**a**) Schematic representation of the experiment set-up. ER were extracted from cells followed by lipids extraction. LC/MS was run to identify the lipidomic profile. (**b**) PCA analysis of lipids extracted from ER show clear separation between U251*^WT^* (blue); U251*^R132H^* (green) and U251*^R132H^* +AGI5198 (red). (**c**) heatmap depicting relative levels of sphingosine, sphinganine, ceramides and downstream ceramide products from U251*^WT^* (blue, *n* = 3); U251*^R132H^* (green, *n* = 3) and U251*^R132H^* + 12.5 µM AGI5198 (red, *n* = 3). (**d**) Schematic representation of sphingolipid pathway highlighting in red the species that are detected in all lipidomic experiments performed. Enzymes are shown in bold and the name of the pathway is underlined. Only ER data are shown since it is the location for the onset of sphingolipid biosynthesis. Serine palmitoyltransferase long chain (SPTLC), 3-ketodihydrosphingosine reductase (KDSR), ceramide synthase (CerS) and dihydroceramide desaturase (DEGS) are enzymes involved in the biosynthesis pathway. Neutral ceramidase (ASAH), alkaline ceramidases (ACER), sphingosine 1-phosphate kinase (SPHK), sphingosine 1-phosphate phosphatase (SGPP) and sphingosine 1-phosphate lyase (SGPL) are part of sphingolipid degradation. Sphingomyelin synthase (SGMS) and neutral sphingomyelinase (SMPD) show the connection between ceramide and sphingomyelin. Ceramide kinase (CERK) is found in the endosome, Golgi and mitochondria; beta-glucocerebrosidase (GBA) and β-1, 4-Galactosyltransferase (β4GalT) are found in the Golgi apparatus and participate in the synthesis of glycosphingolipids that are then stored in lysosomes, which were included to provide a brief overview of downstream ceramide pathways involving other organelles.

To validate out lipidomic findings in the patient-derived cell lines, we used a model system in which U251 glioblastoma cells were genetically engineered to overexpress the IDH1-R132H, the most glioma-prevalent variant of IDH. We treated the engineered IDH1*^mut^* cell line with AGI5198 inhibitor similar to the patient-derived cell lines. In order to measure the alterations in the de-novo sphingolipid synthesis, we extracted the endoplasmic reticulum from cells containing U251*^WT^*, U251*^R132H^* and U251*^R132H^* + AGI5198, and then extracted the lipids from these organelles (Figure 2a).

Lipidomic analysis revealed that there was well-defined separation between each cohort, presenting a progression from U251*^WT^*, U251*^R132H^* treated with IDH*^R132H^* protein inhibitor, to U251*^R132H^* (Figure 2b). Moreover, the introduction of the R132H variant to IDH1 protein resulted in elevated ceramides (especially containing C18:1-fatty acid chains), N-palmitoylserine, sphingomyelin, in addition to glycoceramides (glucosylceramides and gangliosides) which are downstream products of ceramide (Figure 2c,d, Table S3). Their global levels were specifically decreased upon addition of 12.5 µM AGI-5198 inhibitor over 72 h, suggesting a correlation between the levels of D-2HG and increased de-novo sphingolipid synthesis (Figure 2c,d).

### 2.3. Key Sphingolipid Enzymes are Upregulated IDH^mut^ Patient Samples and Cell Lines

**Figure 3:**
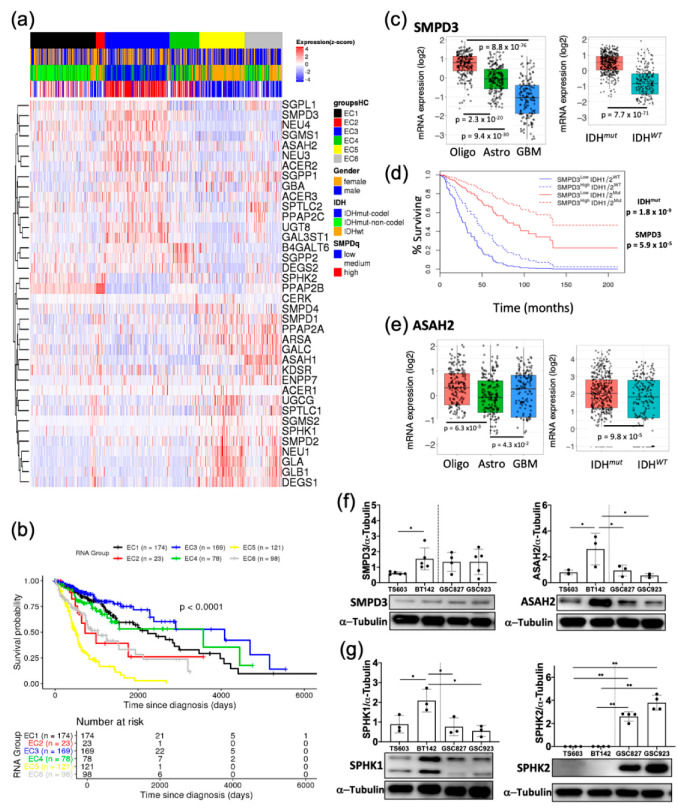
TCGA and Western blot analysis revealed proteins in sphingolipid pathway upregulated in IDH*^mut^* glioma and correlated with increased survival. (**a**) Consensus clustering of genes from sphingolipid pathway using the mRNA levels from the TCGA cohort. The heatmap illustrates gene expression profiles as clustered by the consensus algorithm using clinical patient data showing 6 major expression clusters: EC1 (black), EC2 (red), EC3 (blue), EC4 (green), EC5 (yellow) and EC6 (grey) as indicated. Cluster labeled EC1 is mainly composed of IDH mutant astrocytoma (green outline); EC3 is mainly composed of the IDH mutant with codeletion of chromosomes 1p/19q (oligodendrogliomas) (blue outline); EC5 is predominantly composed of the heterogenous group representing a high influence of IDH^WT^ samples. In the second row, below the EC groups, the gender is represented as female (orange) and male (blue). The third raw histological group is represented as IDH*^mut^* non-codel (astrocytoma, green), IDH*^mut^* 1p/19q codeleted (oligodendroglioma, blue) and IDH*^WT^* (mostly glioblastomas, orange). The last raw depicts the average expression of SMPD2 and SMPD3. (**b**) Kaplan Meier overall survival analysis was applied unsupervised to assess the differences in survival based on primary gene expression among the cohorts as determined by the clustering analysis, and overall Log rank test *p*-value was found to be <0.0001. (**c**) mRNA levels of SMPD3 across the TCGA histological groups and as a function of IDH mutation. (**d**) Kaplan Meier overall survival analysis of patients with high expression of SMPD3 and presence or absence of IDH mutation. (**e**) mRNA levels of ASAH2 across the TCGA histological groups and as a function of IDH mutation. (**f**) Western blot analysis of SMPD3 and ASAH2 in IDH*^mut^* and IDH*^WT^* cell lines. (**g**) Western blot analysis of SPHK1 and SPHK2 in IDH*^mut^* and IDH*^WT^* cell lines. Figure S3: Full WB blot images from Figure 3f,g. Gels were quantified using Image J. T-tests were run in Prism 8.2.1. *p*-values are represented as follows: ** *p* < 0.005; * *p* < 0.05; ns, not significant comparisons were not shown for clarity.

To begin dissecting the role of the sphingolipid pathway to glioma biology, we first looked at the overexpression prevalence of key enzymes of this pathway across the large cohort of patient samples deposited in The Cancer Genome Atlas (TCGA) database. We performed consensus clustering using mRNA levels from 44 genes and 451 samples using R’s TCGA biolinks library. The consensus clustering of the samples was based on the expression of the sphingolipid genes in an unsupervised manner (Figure 3a).

Interestingly, certain enzymes isoform in the sphingolipid pathway were highly expressed and grouped in oligodendroglioma patients (Cluster EC3), while their expression was low in IDH*^WT^* samples (Cluster EC5). By contrast, other isoforms of these enzymes were overexpressed in IDH*^WT^* more (Figure 3a). We focused here on the isoforms overexpressed in IDH1*^mut^* since we are investigating the upregulation of the sphingolipid pathway in this group of gliomas. Such examples are neutral sphingomyelin phosphodiesterase 3 (SMPD3), as well as neutral ceramidase (ASAH2), which appeared amongst the top upregulated enzymes, together with sphingosine-1-phosphate lyase 1 (SGPL1). SMPD3 hydrolyzes sphingomyelin from the lipid rafts to release ceramide while ASAH2 participates in the catabolism of ceramide to form sphingosines and fatty acids. Overexpression of these enzymes has been reported to potentiate stress-induced ceramide accumulation and subsequent caspase-3-directed apoptosis in certain cancers [24,25]. Interestingly, the survival of cluster EC3 was the highest while the survival of a cohort from cluster EC5 was the lowest (Figure 3b). The constitution of these clusters is very different: while cluster EC1 is comprised of IDH1*^mut^* samples that are mostly astrocytoma, the EC3 is formed by oligodendroglioma tissue, and the most aggressive cluster, EC5, is enriched in IDH*^WT^* samples (Table S4). This histological clustering of genes directed us to take a closer look at the individual expression of most upregulated enzyme isoforms in this heatmap, as a function of their histological group or IDH mutated status. We also compared the mRNA levels for SMPD3, ASAH2 and other sphingolipid genes available through the TCGA database between IDH*^mut^* (*n* = 218) and IDH*^WT^* (*n* = 68) from lower grade glioma and higher-grade glioblastomas. As expected, GBM samples had lower population of IDH*^mut^* representatives (*n* = 30) compared with IDH*^WT^* (*n* = 372). Patients’ samples with IDH*^mut^* consistently displayed significantly higher mRNA levels of SMPD3 and ASAH2 along with related genes in both lower grade glioma as well as high grade gliomas (Figure 3a,c–e). To account for the fact that IDH*^mut^* contributes positively to patient cohort survival, we then separated the samples according to IDH mutation and computed the survival of those four groups again. Interestingly, amongst all of the highly expressed genes in this pathway, SMPD3 overexpression was associated with improved survival in both IDH*^mut^* and IDH*^WT^;* however, it was more significant in IDH1*^mut^* lower grade gliomas (*p* value of 1.2 × 10^−8^ for IDH*^mut^* compared with 1.7 × 10^−3^ for IDH*^WT^*) (Figure 3d). Overall, certain sphingolipid genes (e.g., SMPD3 and ASAH2) follow a similar pattern; their mRNA levels are very high in oligodendroglioma, followed by astrocytoma, and finally the lowest in glioblastomas (Figure 3c,e). These analyses point to upregulation of certain genes in the sphingolipid degradation pathway that lead to an accumulation of ceramides and sphingosine in IDH1*^mut^* glioma and most importantly correlate with the patient samples with our in vitro metabolomics discovery.

Next, we verified that the genes upregulated in the TCGA cohort of patients are also upregulated in our cellular models. We chose TS603 to be representative of oligodendroglioma and BT142 cell lines from the astrocytoma histological group, while GSC827 and GSC923 cell lines were representatives of IDH1*^WT^* glioblastomas. Indeed, a comparison of SMPD3 and ASAH2 proteins levels in these cell lines show an upregulation of the sphingolipid degradation pathway in both oligodendroglioma as well as astrocytoma (Figure 3e,f, Figure S1). Interestingly, the astrocytoma cell line available to us (BT142) presented higher expression of SMPD3 and ASAH2 compared with the oligodendroglioma. Moreover, sphingosine kinase isoforms SPHK1 and SPHK2, the enzymes involved in sphingosine 1-phosphate formation, displayed noteworthy expression in our available cell lines. The BT142 cell line displayed the highest expression of SPHK1 protein amongst all cell lines including GBM. In contrast, SPHK2 was highly expressed in glioblastoma cell lines (GSC827 and GSC923) while it was undetectable in both IDH1*^mut^* cell lines (Figure 3g, Figure S1). Overall, Western blot analysis confirmed upregulation of key sphingolipid enzymes such as SMPD3 and ASAH2, in IDH1*^mut^* glioma cells specifically, which suggested that the pathway is upregulated towards the elevating the availability of sphingosine and ceramide.

### 2.4. Targeting the Sphingolipid Pathway in IDH1^mut^ Glioma Cells

**Figure 4:**
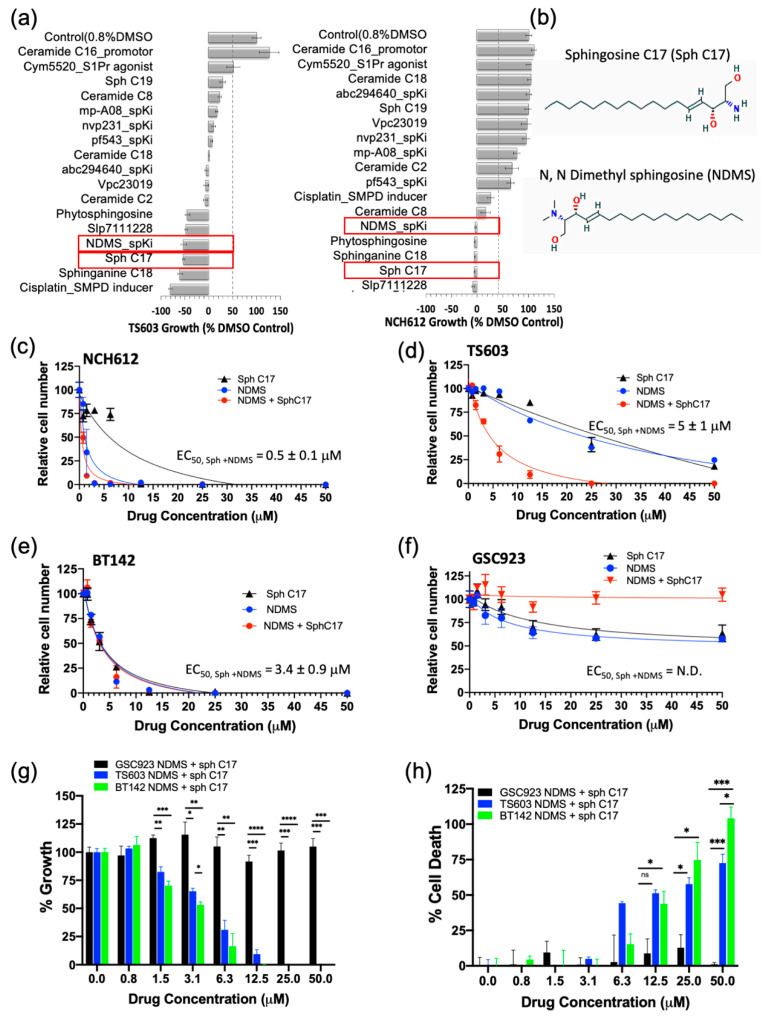
Modulating the sphingolipid pathway induces IDH*^mut^*-specific cell death. (**a**) Rational drug screen to include 17 bioeffector molecules and inhibitors of sphingolipid pathway for two oligodendroglioma cell lines (TS603 and NCH612). Red rectangles highlight the molecules chosen to be analyzed further. (**b**) Chemical structures of the most active sphingolipids resulted from the screen sphingosine C17 and N, N dimethyl sphingosine. (**c**–**f**) EC_50_ measurements of three IDH*^mut^* glioma and one IDH*^WT^* glioblastoma cell line. The EC_50_ measurements were obtained via fitting to the equation from Graph Prism software. N.D, not determined. (**g**) Side-by-side comparison of growth rates between IDH*^WT^* (GSC923, black) and IDH*^mut^* (BT142, green; TS603, blue) cell lines treated with the drug combination of Sph C17 and NDMS. The data were obtained using a CCK8 assay described in the Methods section and plotted with Graph Prism 8.2.1. (**h**) Side-by-side comparison of percent cell death between IDH*^WT^* (black) and IDH*^mut^* cell lines (BT142, green; TS603, blue) treated with the drug combination of Sph C17 and NDMS. The data were obtained using a CCK12 assay described in the Methods section and plotted with Graph Prism 8.2.1. Statistical analysis (t-test) was conducted assuming equal variance, *p*-values are represented as follows: **** *p* < 0.00005, *** *p* < 0.0005; ** *p* < 0.005; * *p* < 0.05; ns, not significant. For simplicity, only significant values are shown on the graph.

Having demonstrated that the IDH1^mut^ glioma cell sphingolipid profile contains high sphingosine/ceramide and low S1P from both metabolite as well as enzyme overexpression analyses, we next targeted the sphingolipid pathway with the goal to determine a combination of compounds that effectively arrests tumor cell proliferation and leads to cell death. We initially tested an inhibitor of sphingosine kinase I (SphK1), PF-543, to demonstrate the feasibility of enhancing this potential metabolic vulnerability by further decreasing intracellular levels of S1P. Morphologically, the BT142 cell line displayed a higher sensitivity to PF-543 as seen in the optical images (Figure S2). Given the promising results of inhibiting SphK1 in two IDH1^mut^ cell lines (BT142 and TS603), we expanded the panel to include additional SphK inhibitors (SpKi) and other sphingolipid-based bioeffectors. Therefore, we designed a small scale, rational drug screen of 17 related compounds including commercially available SphK inhibitors as well as specific endogenous bioeffectors, such as NDMS, sphingosine-C17 (Sph C17) and sphinganine-C18 (dhSph C18), which were determined to be elevated in our metabolic analysis of IDH1^mut^ neurospheres. By treating IDH1^mut^ cells with these sphingoid bioeffectors, we rationalized that this approach would further enhance the metabolic/cellular stress already existent due to their intrinsic elevation in IDH^mut^ glioma lines. In the rational drug panel, we also included ceramide C16, which was previously reported to promote growth [19] as well as cisplatin, which was reported to activate acid sphingomyelinase (SMPD2) [26]. Amongst the 17 compounds, NDMS, sphingosine-C17 and sphinganine-C18 were consistently effective and potent in both representative IDH1^mut^ glioma cell lines (Figure 4a,b). Interestingly, NDMS, which is an endogenous bioeffector that inhibits SphK, elicited a potent biostatic effect that exceeded most of the synthetic SphK inhibitors present in our rational panel, particularly PF543 used in the pilot experiment.

Since the elevation of endogenous bioeffectors, NDMS and sphingosine C17 elicited a highly potent biostatic response in two IDH1^mut^ cell lines, we decided to determine their dose-dependent effects on cell viability (proliferation) and cytotoxicity (cell death). Both the cell proliferation and cell death assays confirmed that all of the tested IDH1^mut^ cells presented a dose-dependent response to treatment with the NDMS and sphingosine and produced a greater effect when combined. In particular, the oligodendroglioma cell lines (TS603 and NCH612) displayed an increased effect by combining both NDMS and Sph C17 while the astrocytoma cell line (BT142) was equally sensitive to independent versus combined treatment (Figure 4c–e, Table S5). By contrast, the IDH1^WT^ cell line (GSC923) presented little sensitivity to these drug or combination treatments (Figure 4f–h, Table S5).

The response to the combinatory treatment suggests high specificity to IDH1^mut^ cell lines both on the growth of the cells (Figure 4g) as well as the percentage of cellular death (Figure 4h). Collectively, these findings suggest a model in which targeting sphingolipid metabolism by elevating sphingosine and inhibiting SPHK with N,N-dimethylsphingosine presents a remarkable vulnerability of IDH1^mut^ gliomas. Further investigation of the downstream sphingolipid signaling events that are disrupted in tandem with this treatment is expected to reveal additional vulnerable targets in the cell-regulation of IDH1^mut^ gliomas.

## 3. Discussion

**Figure 5:**
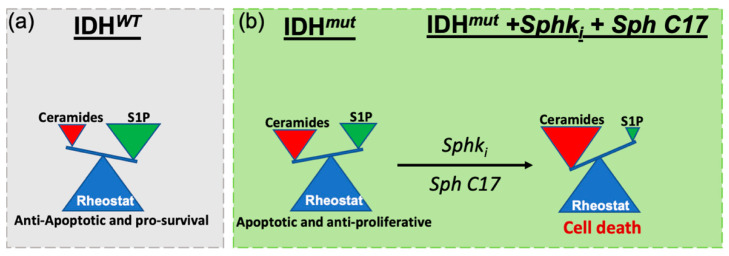
Proposed model for the specific IDH*^mut^* cell death induced via the modulation of cellular levels of sphingosine 1-phosphate and ceramides. (**a**) In IDH*^WT^* the ceramide to S1P rheostat is tilted towards high S1P levels which stimulate cellular signaling for anti-apoptotic and pro-survival. (**b**) IDH*^mut^* glioma present a reverse in the sphingolipid rheostat such that high ceramide and low S1P levels are present. Further exacerbation of this rheostat balance via inhibition of SphK and addition of sphingosine C17 (a reversible, immediate metabolite of ceramide) triggers cellular arrest and death in IDH*^mut^* gliomas.

The limited improvement in the overall survival of glioma patients highlights the crucial need for effective therapeutic strategies against these aggressive tumors. The discovery of prevalent mutations in IDH1 has revealed a unique metabolic phenotype in these tumors and motivated us to explore metabolic-based drug targets via lipidomic-based workflows. Earlier investigations have exhaustively explored ways to target central carbon metabolism [7]. However, few studies have explored alteration in the lipidome that are relevant for IDH1*^mut^* gliomas [22,27]. Our laboratory has begun to unravel unique perturbations along lipid pathways that are associated with IDH1 mutations and offer potential avenues for translation to therapeutic targets [22]. Herein, we reported for the first-time, to our knowledge, dysregulations in sphingolipid metabolism as they relate to IDH*^mut^* gliomas. In particular, we identified decreased levels of S1P and increased levels of ceramide, sphingosine and sphinganine that correlate with the presence of IDH1 mutation. We validated this sphingolipidomic profile using gene expression and Western blot analyses, which suggested that overexpression of key enzymes, SMPD3, SGPL, ASAH2 (ceramidase/sphingosine synthase) and S1P phosphatase (SGPP2) that tilt sphingolipid rheostat toward increased ceramide and decreased S1P are associated with IDH*^mut^* gliomas, while not within IDH*^WT^*. Based upon these findings, we proposed a working model in which the sphingolipid rheostat between ceramides and S1P is inverted in IDH*^mut^* glioma compared with IDH*^WT^* counterparts (Figure 5a,b).

To exploit this funding therapeutically, we employed a rational drug panel screen, which revealed that modulating the sphingolipid rheostat towards accumulation of sphingosine and blocking the synthesis of S1P, we could induce cellular death in a dose-dependent manner IDH*^mut^* gliomas subtypes. Ultimately, these findings suggest that IDH*^mut^* tumors are susceptible to the manipulation of sphingolipid-related metabolism and signaling. While the contribution of altered sphingolipid signaling to glioblastoma malignancy and progression is well-documented, and the properties of altering the sphingolipid rheostat to favor elevation of S1P have been linked to all the hallmarks of glioblastomas [28], its role in IDH*^mut^* glioma remains unexplored. To the best of our knowledge, this is the first study that shows alteration in sphingolipid metabolism specific to lower grade gliomas with an IDH mutation and ways to exploit this intrinsic vulnerability. Unraveling this unique vulnerability in lower grade gliomas with IDH mutation provides avenues for future therapeutic strategies. Future investigations will expand on the mechanisms by which this combinatorial treatment trigger cellular death, the signaling involved, as well we the preclinical evaluation of these metabolites.

## 4. Materials and Methods

### 4.1. Cell Culture and Sample Collection

Media for specific human-derived stem-like spheroids (BT142, BT142+ AGI5198, TS603, TS603+ AGI5198, GSC923) was prepared using 500 mL DMEM: F12 media (Gibco Laboratories, Gaithersburg, MD, USA) with the following additives: 5 mL penicillin/streptomycin 100×, 5 mL N_2_ growth supplement 100×, 100 µL epidermal growth factor (EGF) and 100 µL fibroblast growth factors (FGF) obtained from ThermoFisher Scientific (Waltham, MA, USA) and 2 µg/mL heparin sulfate (Millipore-Sigma, Burlington, MA, USA). The human derived NCH612 glioma cell media was prepared using 500 mL DMEM: F12/Glutamax media with the following additives: 5 mL Penicillin/Streptomycin 100×; 100 µL EGF and FGF; 10 mL B27 supplement 100× (ThermoFisher Scientific). Following the addition of additives, the media was filter-sterilized and store at 4 °C. Media for 72 h treatment with IDH1^R132H^ inhibitor were prepared at a concentration of 50 µM AGI5198. Cells phenotype was validated based upon DNA methylation (1p/19q co-deletion) as previously described [29], DNA sequencing (IDH1 mutation) [29] and D-2HG measurements (IDH1 mutation) [22].

Nonadherent cell spheroids were grown in triplicate to a minimum density of 3 million cells per flask. Each sample flask received 1 mL of media and allowed to grow for 72 h at 37 °C. Samples were transferred to 15-mL pre-sterilized conical tubes and centrifuged at 400× *g* for 5 min at room temperature. Then, media from supernatant was collected. Cell pellets were washed with 600 µL phosphate-buffered saline (PBS) and centrifuged at 400 rpm for 5 min at room temperature (RT). Supernatant was discarded. Pellets were snap-frozen on dry ice and stored at −80 °C to ensure complete quenching of all metabolic activity and degradation until extraction. Adherent cells were grown in triplicate to a confluence ≥90% in a large-sized T175 tissue culture flask. About 20 flasks were used per variant (U251*^WT^*, U251*^R132H^*, U251*^R132H^* + 12.5 μM AGI5198). Prior to collection, media was collected. Each flask was administered 3.0 mL 0.05% Trypsin and incubated for 1 min. Pipetting was conducted to dissociate adherent cells from plate surfaces. The homogenous cell media was transferred to sterile conical tubes and centrifuged at 400× *g* for 5 min at room temperature. Supernatant was discarded and pellet was washed with 1.0 mL PBS. Cells were centrifuged 400× *g* for 3 min at room temperature. Supernatant was completely aspirated and discarded. Approximatively 200 mg of wet cell pellet was used for the extraction of endoplasmic reticulum using ER extraction kit from Invent Biotechnologies (Plymouth, MN, USA) according to the manufacture instructions, as previously described [22]. Pellets containing ER were snap frozen and stored at −80 °C until extraction.

### 4.2. Metabolite Extraction

Prior to sonication, tissue and cells samples were administered 400 µL ice-chilled MilliQ water and lysed via sonication by Misonix XL-2000 Ultra-liquid processor (Misonix Inc., Farmingdale, NY, USA) at 40 amps for 30 s. Following sonication, an aliquot (5% total volume) of each cell lysate was collected for Bradford protein assay to later normalize target analyte concentration to nmol/mg protein. Prior to extraction, 100 µL IS solution containing 0.05 μg/mL 3-phenyl-N-(4-pyridinyl)acrylamide (PNPA) in chloroform and 100 µL IS solution containing 0.125 μg/mL *p*-Nitrobenzoic acid (NBA) and 0.125 μg/mL debrisoquine sulfate (DBQ) in 60% MeOH (aq) was added to each lysate. Sample was vortexed on BenchMixer (Benchmark Scientific, Edison, NJ, USA) at mid-speed (6) for 15 s and incubated in ice for 20 min on rotating mixer at mid-speed.

Upon extraction, 600 µL chilled (−20 °C) LC/MS grade Methanol (MeOH) was added to each 380 µL sample lysate spiked with IS. Samples were vortexed with for 30 s and placed on Orbi-blotter mixing rotator (Benchmark Scientific, Edison, NJ, USA) at max speed to incubate in ice-bath for 10 min. Samples were vortexed and administered 300 µL chilled (−20 °C) chloroform to each sample and returned to ice-bath on rotator for 60 min to precipitate protein. All samples remained in ice-bath until final centrifugation and collection. Samples were centrifuged at 13,000× *g* for 20 min at 4 °C. The resulting two phases (upper hydrophilic and lower hydrophobic lipid) were separated while the remaining protein disk layer was discarded. Extracts were concentrated under N_2_ gas flow on Techne sample concentrator with PTFE-coated needles (Cole-Palmer, Vernon Hills, IL, USA) at 25 °C until completely dry, snap frozen and then stored at −80 °C.

### 4.3. Biostatic Drug Screening

The rational screening to target and alter sphingolipid pathway utilized the CCK8 proliferation assay described below. For the drug screening with CCK8, samples were treated at 50 µg/mL per candidate in triplicate and incubated for 48 h in CO_2_ incubator (37 ℃). Drug efficacy was assessed based on the degree of the biostatic response compared to the proliferation measurement detected for non-treated 0.8% DMSO control. Candidates presenting growth inhibition ≥50% were considered a hit. Candidates with negative values were interpreted as negligible or no growth as the mean absorbance was below the blank control composed of DMSO and media only.

### 4.4. Cell Proliferation Assay (CCK-8)

In order to test the effects of the drug treatment on proliferation, added appropriate media (50 μL) was prepared with drug at the desired concentration for final well volume (110 μL) at 0.8% *v/v*. The density of cell suspensions was diluted appropriately (based on optimization experiment) then wells were inoculated with 50 μL cell suspension in Corning sterile 96-well non-treated black chimney, clear round bottom plate for spheroids (Millipore-Sigma). CCK-8 solution (10 μL) was added to each well of the plate. Plates were covered with a lid and placed in cell CO_2_ incubator at 37 ℃. Each plate was incubated for 24 h in the incubator prior to measuring the absorbance at 450 nm and 515 nm using a microplate reader. The blank controls were included with media and 0.8% DMSO to subtract background from each well, and the corrected mean absorbance was determined for each set of triplicates.

### 4.5. LDH Cytotoxicity/Cell Death Assay (CCK-12)

In order to test the cytotoxic effects of the drug treatment, added appropriate media (50 μL) prepared with drug at desired concentration for final well volume (110 μL) at 0.8% *v/v*. The density of cell suspensions was diluted appropriately (based on optimization experiment) then 60 μL cell suspension was added to each well in the Corning sterile 96-well non-treated black chimney, clear round bottom plate for spheroids. Plates were covered with lid and placed in cell CO_2_ incubator at 37 ℃. The non-lysed control (70 h), and two lysed controls at 2 h (low), and 70 h (high) were prepared in triplicate. The Lysis buffer (10 μL) was added to each well for high control 1 h prior to measuring at the 2 h and 72 h timepoints, respectively. Then, plates were covered with a lid and returned to the CO_2_ incubator at 37 ℃. Working Solution (100 μL) was added to each well 30 min prior to reading. The lid was replaced, the plate was wrapped in foil to protect from light, and incubated at the room temperature for 30 min. Stop Solution (50 μL) was added to each well and absorbance measured at 490 nm by a microplate reader. The blank controls were included and composed of media + 0.8% DMSO to subtract background from each well, and the corrected mean absorbance was determined for each set of triplicates.

### 4.6. LC/MS Experimental Set-Up

LC/MS lipidomic analysis was acquired on the Agilent 6545 Quadrupole Time-of-Flight Mass Spectrometer coupled with Infinity II 1290 Liquid Chromatography Ultra-High-Pressure system (Agilent Technologies Inc., Santa Clara, CA, USA).

#### 4.6.1. Organelle Specific Lipidomic Methods for ER

Organelles were extracted as previously described [22]. The hydrophobic phase from ER extracts were reconstituted in 100 µL reagent containing 3:2:1:4 EtOH/IPA/ACN/water (aq). Pooled quality control (QC) samples were composed with 10% volume of each sample. Lipids were resolved using Acquity UPLC CSH 1.7 µm, 2.1  ×  100 mm column (Waters Corp. Milford, MA, USA) with a gradient described previously [22,30].

#### 4.6.2. Sphingolipid and Polar Lipid Optimized Methods

Prior to analysis, hydrophilic and hydrophobic phases were reconstituted and combined with 100 µL 4:2:4 EtOH/MeOH/water. Metabolites were resolved using a coupled column LC method to resolve and retain polar lipids including sphingolipids by incorporation of HILIC Xbridge BEH amide 2.5 µm, 2.1 × 50 mm column xp (Waters Corp) coupled with a Pentafluorophenyl (PFP) InfinityLab Poroshell 120, 1.9 µm, 2.1 × 100 mm (Agilent Technologies Inc.) utilizing a gradient composed of mobile phase A, 30 mM ammonium acetate (NH_4_Ac) (aq) + 0.1% FA + 0.015% Infinity Lab Deactivator (Agilent Technologies, Inc.); and mobile phase B, 5:10:85 30 mM NH_4_Ac/MeOH/ACN at pH 4.3. An isothermal column temperature of 40 °C and static flow rate of 0.200 mL/min was maintained during the following gradient timetable: 0–1.5 min, 2% B; 3.5 min, 67% B, 5.5 min 100% B; hold 1.5 min; 8 min, 75% B; 8.5 min, 100% B; hold 0.75 min; 11.5 min, 2% B, equilibrate, 1.5 min. Real-time mass correction was applied with 0.2 mL/min infusion of external standard (containing TFA/PURINE/HP921) in 95:5 ACN/water. Electrospray injection (ESI) negative ion acquisition was applied with the following MS parameters: injection volume, 7.5 µL; drying gas temperature (temp), 250 °C; drying gas flow, 8 L/min; nebulizer pressure, 40 psi; sheath gas temp, 350 °C; sheath gas flow, 12 L/min; capillary voltage, 3000 V; nozzle voltage, 15 V; fragmentor, 90 V; skimmer, 50 V; scan rate, 3.0 spectra/s; mass range 75–1300 m/z. Alternatively, ESI positive ion acquisition applied the following MS parameters: injection volume, 6.5 µL drying gas temperature (temp), 250 °C; gas flow 8 L/min; nebulizer, 40 psig; sheath gas temp, 350 °C; sheath gas flow, 12 L/min; capillary voltage, 3500 V; nozzle voltage, 15 V; fragmentor, 170 V; skimmer, 50 V; scan rate, 4.0 spectra/s; mass range, 75–1300 m/z.

#### 4.6.3. LC/MS Data Processing and Statistical Analysis

Prior to preprocessing each dataset, pooled QC samples (TIC, BPI and EIC) were chromatographically examined to inspect consistency of retention time and ionization levels throughout. Following acquisition, mass feature bins were defined by partitioning the *m/z* vs. retention time (RT) matrices into a fixed width using Agilent Masshunter Profinder B.08.00. Bins were manually inspected to confirm consistent, reproducible integration for each compound of interest across all samples. Precursor m/z for each bin was determine using a molecular feature extraction algorithm to deconvolute, integrate and envelope parent ions, adducts (H^−^, Cl^+^, H^+^, Na^+^), natural isotopes and neutral losses to define each composite spectrum. Targeted ion selection, alignment and annotation for logical binning of the input data were restricted to ion mass accuracy ±5.0 mDa and retention time ±0.4 min using an in-house Personal Compound Data Library (PCDL). Following pre-processing, the ion abundance for each sample was corrected using sample-specific weight quantification. Biostatistical analysis of LC-MS profile data was performed using Metaboanalyst 4.0 software [23]. Values were corrected to sample-specific internal standard abundance and protein mass, then normalized to the median to perform statistical analysis with equal variance t-test for binary comparisons and Fisher’s LSD post-hoc ANOVA for multiple group comparisons.

### 4.7. TCGA Analysis of Sphingolipid Pathway

Cox proportional hazards regression analysis on overall survival were performed on Lower Grade Glioma data generated from The Cancer Genome Atlas (TCGA) downloaded from cBioPortal (http://www.cbioportal.org/) and Firehose (https://gdac.broadinstitute.org/) using R’s libraries cgdsr and RTCGA Toolbox. RNA-Seq mRNA expression data were downloaded using R’s TCGA biolinks library. In all, 672 primary glioma samples (516 LGGs, 156 GBMs) were preprocessed, normalized, and filtered as recommended in R’s TCGA Workflow. Unsupervised consensus clustering of the samples was performed on the expression of the 38 (sufficiently expressed) genes from the Sphingolipid Metabolism pathway from the KEGG (https://www.genome.jp/kegg/) collection. Clustering into 6 groups was considered, with clustering into 4 groups assessed as falling in the optimal range. Composition of the groups in terms of TCGA glioma subtyping is shown in the table. Kaplan Meier overall survival analysis was performed using TCGA biolinks’ survival analysis function. Pairwise survival differences between the groups were assessed using the survival library of R and the *p*-values were adjusted for multiple comparisons using FDR correction.

R’s survival package was used to perform Cox proportional hazards ratio analysis on overall survival using the IDH-mutation status and expression of the SMPD3 gene as covariates. Survival curves for the IDH mutant case were plotted using expression of SMPD3 at 25% and 75%. In total, 451 samples with known IDH1/IDH2 mutation status (366 IDH*^mut^*, and 85 IDH^WT^) and available RNASeq mRNA expression data were used in the analysis. Cox proportional hazards regression analysis on overall survival were performed on Lower Grade Glioma data generated from The Cancer Genome Atlas (TCGA) downloaded from cBioPortal (http://www.cbioportal.org/) and Firehose (https://gdac.broadinstitute.org/) using R’s libraries cgdsr and RTCGA Toolbox.

### 4.8. Western Blot Analysis

The cellular proteins were purified from treated or control cells. Equal amounts of protein (10 μg) were loaded in each lane for NuPAGE 4–12% Bis-tris gel and then transferred to polyvinylidene difluoride membranes. The membranes were washed with blotting buffer (1× PBS containing 0.1% Tween20) and then blocked for 60 min in blotting buffer containing 10% low-fat powdered milk. Membranes were washed 3 times with blotting buffer, incubated at 4 °C overnight with primary antibody (1:1000) containing 5% low fat powdered milk and incubated with HRP conjugated secondary antibody (1:1000) at room temperature for 60 min. The blots were detected with Bio-Rad image system. The relative expression of proteins was normalized to a-Tubulin (cat.no: ab7291), and analyzed using Image J. ASAH2 (cat.no: ab170949), SGPP1 (cat.no: ab108435), SPHK1 (cat.no: ab109522), SPHK2 (cat.no: ab264042), SMPD2 (cat.no: ab131330), SMPD3 (cat.no: ab172193) were purchased from Abcam. SGPP2 (cat.no: PA5-42767) was purchased from Thermo Fisher.

## 5. Conclusions

In this study, we revealed that a novel combination of bioeffectors from the sphingolipid pathway could be administered to selectively inhibit proliferation and trigger cell death in IDH1*^mut^* glioma cell lines. Ultimately, the response of IDH1*^mut^* gliomas to the targeting of SphK1 in order to obstruct S1P production and subsequent S1P signaling suggests that the sphingolipid metabolism plays a vital role in glioma’s growth and survival. Given that ceramide, sphingosine and S1P are reported to maintain a rheostat balance, we postulate that inhibiting the production of S1P might trigger an accumulation of ceramide as well as intermediate sphingosine and directly impact on tumor survival [21]. The discovery of the sphingolipid pathway dysregulation in IDH1*^mut^* gliomas, described herein, provides new avenues for further evaluation in preclinical models and potential therapeutic interventions in the future.

## Supplementary Materials

The following are available online at https://www.mdpi.com/2072-6694/12/10/2910/s1, Figure S1: Western Blots for ASAH2, SMPD3, SPHK1, SPHK2 and a-Tubulin; Figure S2: The effect of SPHK1 inhibitor PF-543 on BT142 and TS603 cell morphology, Figure S3: Full WB blot images from Figure 3f,g, Table S1: Sphingolipids and polar lipid analysis for BT142 untreated and treated with AGI5198, Table S2: Sphingolipids and polar lipid analysis for TS603 untreated and treated with AGI5198, Table S3: ER sphingolipids analysis for U251*^WT^*, U251*^R132H^* and U251*^R132H^* + AGI5198, Table S4: Histological and Methylation Types of Tissue Present in Clusters EC1-EC6, Table S5: EC50 for SphC17, NDMS and combination treatments.
