# Supplementary material for: Sphingolipid Pathway as a Source of Vulnerability in IDH1mut Glioma
Source: Cancers (Basel). 2020 Oct 10;12(10):2910. doi: 10.3390/cancers12102910 (PMC7601159; doi:10.3390/cancers12102910)
Supplement: Supplementary file 1 [file cancers-12-02910-s001.pdf]

Supplementary Material: Sphingolipid Pathway As a Source of Vulnerability in IDH1^{mut} Glioma

Tyrone Dowdy, Lumin Zhang, Orieta Celiku, Sriya Movva, Adrian Lita, Victor Ruiz-Rodado, Mark R. Gilbert and Mioara Larion *

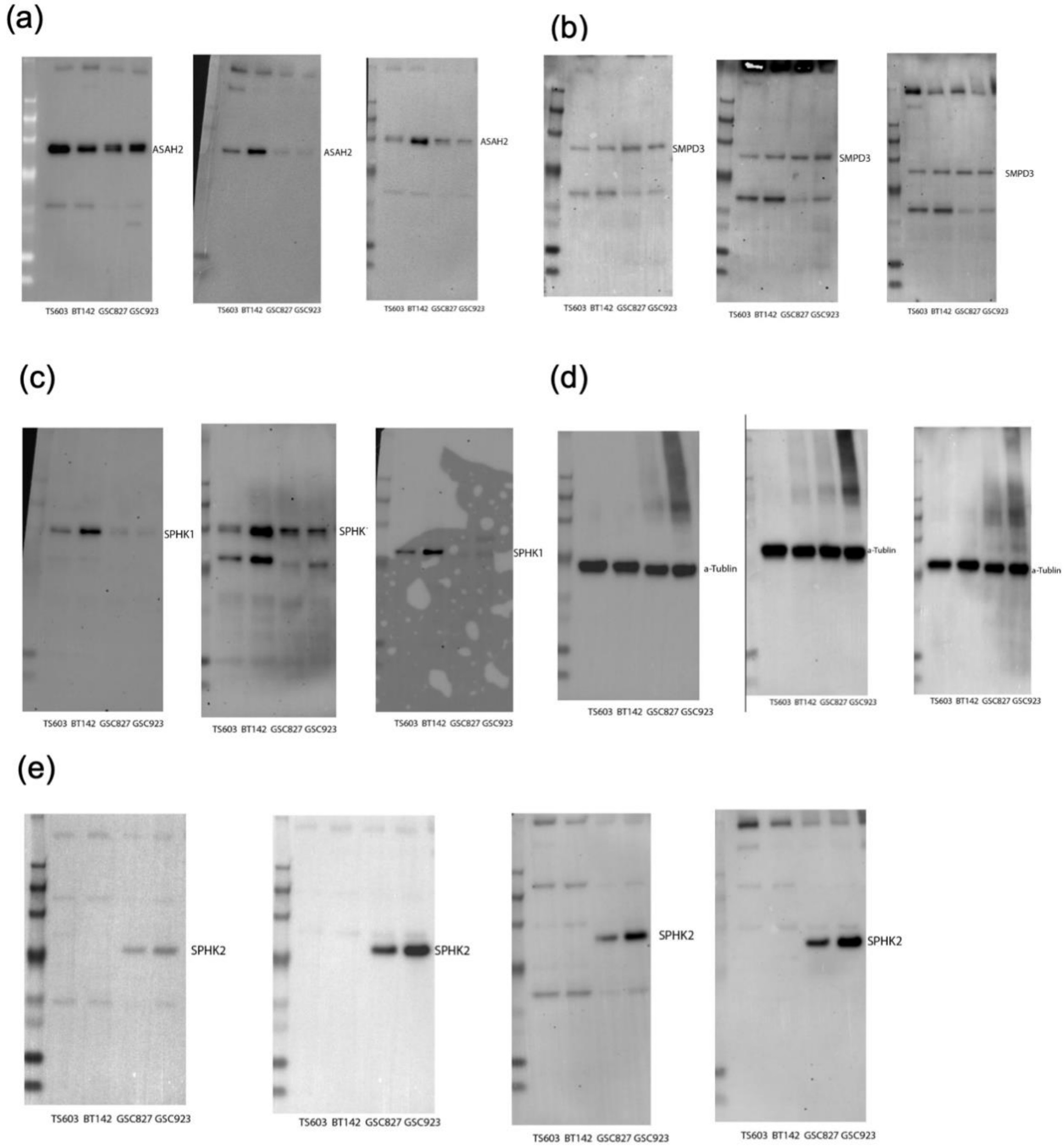

Figure S1. Western Blots for ASAH2, SMPD3, SPHK1, SPHK2 and a-Tubulin.

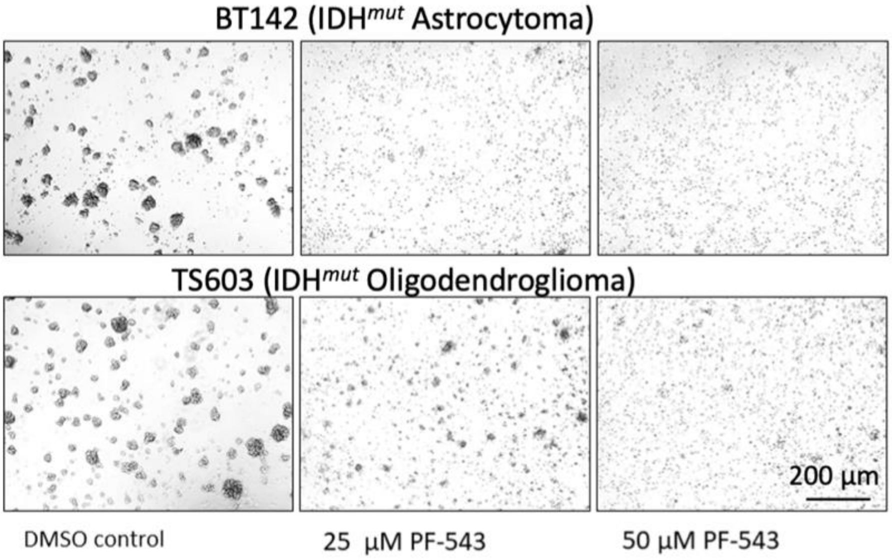

Figure S2. The effect of SPHK1 inhibitor PF-543 on BT142 and TS603 cell morphology.

Table S1. Sphingolipids and polar lipid analysis for BT142 untreated and treated with AGI5198.

ID	FC	raw. pval	FDR
PAF C-18	5.19	1.90E-04	9.58E-03
Palmitolinoleic acid	2.65	2.01E-04	9.58E-03
S-aminomethyldihydrolipoamide	3.97	2.12E-04	9.58E-03
Sphingosine 1-P	0.18	2.66E-04	9.58E-03
PIP 16-1,16-1	0.14	2.72E-04	9.58E-03
N-Arachidonoylglutamine	0.03	2.87E-04	9.58E-03
LPC 14-0	0.01	3.48E-04	9.58E-03
Tetrahydrocorticosterone	0.09	3.51E-04	9.58E-03
LPC 18-0	3.22	3.54E-04	9.58E-03
4-Ketopalmitic acid	3.14	3.55E-04	9.58E-03
N-Arachidonoyltyrosine	0.03	4.01E-04	9.58E-03
C17 Sphinganine	12.47	4.11E-04	9.58E-03
N-Arachidonoylsphinganine	0.21	4.21E-04	9.58E-03
Glutaminyl-Arginine	2.87	4.40E-04	9.58E-03
Palmitoylglycerophosphocholine	11.86	5.16E-04	1.00E-02
LPC 16-0	11.91	5.26E-04	1.00E-02
LPC 22-5	0.13	6.74E-04	1.18E-02
Dehydrophytosphingosine	2.87	6.97E-04	1.18E-02
Sulfatide C23-0	0.18	9.55E-04	1.53E-02
Sulfatide Ch24-1	0.23	1.01E-03	1.55E-02
L-Leucine	0.11	1.12E-03	1.63E-02
PGF2-ethanolamine	11.98	1.27E-03	1.75E-02
Kynurenine	3.36	1.32E-03	1.75E-02
Eicosenoic acid	2.68	2.14E-03	2.72E-02
oxo-Resolvin D1	3.79	2.74E-03	3.34E-02
9R-HOME	3.87	3.06E-03	3.59E-02
L-Homotyrosine	2.32	3.51E-03	3.96E-02
Sphingosine C17	2.31	3.67E-03	4.00E-02
Palmitoleic acid	2.78	4.21E-03	4.38E-02
N-Stearoylserine	82.93	4.30E-03	4.38E-02
CerP d18-1,8-0	3.14	4.49E-03	4.41E-02
Sphingosine C18	2.47	5.12E-03	4.83E-02
3-Ketosphinganine	2.50	5.23E-03	4.83E-02
Phytosphingosine 1-P	0.11	6.02E-03	5.40E-02
HpOME	4.60	6.44E-03	5.61E-02

O-3-Arachidonate	3.32	7.06E-03	5.94E-02
Coenzyme Q4	2.17	7.33E-03	5.94E-02
Cystine	1.83	7.40E-03	5.94E-02
N-Oleoyltaurine	6.34	7.72E-03	5.97E-02
5-Ketopalmitic acid	2.47	7.83E-03	5.97E-02
O-D-glucopyranuronosyl-N-tetradecanoyldihydrosphingosine	3.49	1.25E-02	0.09
Sphinganine C24-1	0.25	1.29E-02	0.09
LPC 18-2	0.34	1.37E-02	0.10
D-glucopyranuronosyl-N-tetradecanoyldihydrosphingosine	2.55	1.44E-02	0.10
N,N-Dimethylsphingosine (NDMS)	2.65	2.22E-02	0.14
11R-HOME	1.92	2.34E-02	0.14
PGD2, 2-glyceryl-	0.19	2.37E-02	0.14
Ubiquinol	1.45	2.87E-02	0.16
Ceramide-1-phoshoethanolamine	0.20	2.87E-02	0.16
LPE 18-0	0.14	3.08E-02	0.17
PGF2-acetate	3.00	3.25E-02	0.17
Sulfatide C25-1	2.50	3.26E-02	0.17
Arachidonoyldopamine	0.01	3.27E-02	0.17
Ubiquinone-4 (Coenzyme Q4)	1.75	3.31E-02	0.17
L-Glutamine	1.83	3.44E-02	0.17
Sulfatide Ch18-0	1.96	3.65E-02	0.18
Sulfatide C26-0	1.26	3.86E-02	0.19
Sulfatide Ch22-0	0.72	4.02E-02	0.19
Hydroxymyristic acid	1.59	5.03E-02	0.23
Eicosatrienoic acid	2.20	5.06E-02	0.23
PIP 16-0,18-0	0.61	5.27E-02	0.23
Palmitic amide	4.92	5.48E-02	0.23
Cer d18-1,16-1	2.03	5.92E-02	0.23
Sulfatide Ch25-1	1.73	5.93E-02	0.23
LacCer d18-1,16-0	1.74	5.94E-02	0.23

Table S2. Sphingolipids and polar lipid analysis for TS603 untreated and treated with AGI5198.

ID	FC	raw.pval	FDR
LPC 14-0	0.00	1.5E-09	4.5E-07
PGF2-ethanolamine	17.27	2.5E-08	3.9E-06
N-Arachidonoylglutamine	0.18	1.2E-07	1.2E-05
PhytoS1P (Phytosphingosine-1-P)	0.10	2.8E-07	2.1E-05
LPI 16-1	0.17	7.7E-07	4.8E-05
S1P (Sphingosine-1-P)	0.10	1.5E-06	7.9E-05
S-aminomethyldihydrolipoamide	7.92	4.1E-06	1.4E-04
N-Arachidonoyltyrosine	0.31	8.8E-06	2.7E-04
N-Oleoyltaurine	2.67	1.3E-05	3.7E-04
PGD2, 2-glyceryl-	0.66	3.2E-05	8.3E-04
9R-HOME	1.82	7.4E-05	1.7E-03
O-3-Arachidonic acid	2.33	8.6E-05	1.7E-03
Kynurenine	2.33	8.6E-05	1.7E-03
Gamma-Glu-Ser	0.39	9.5E-05	1.7E-03
Glutaminyl-Ser	0.39	9.5E-05	1.7E-03
HpOME	2.28	1.1E-04	2.0E-03
Dehydrophytosphingosine	1.91	1.4E-04	2.2E-03
NDMS (N,N-Dimethylsphingosine)	2.00	1.5E-04	2.3E-03
Palmitic amide	2.05	1.6E-04	2.4E-03
PIP 16-1,16-1	0.10	1.7E-04	2.4E-03
Oleamide	2.19	3.0E-04	4.1E-03
Oleoyl-EA	2.42	3.2E-04	4.1E-03
Eicosenoic acid	1.86	5.7E-04	7.2E-03
Sphinganine C17	2.14	7.6E-04	9.1E-03

Arachidonoyl-PI (glycerophosphomyoinositol)	3.13	9.1E-04	1.0E-02
Glutaminyl-Arg	2.01	1.0E-03	1.1E-02
Palmitolinoleic acid	1.76	1.1E-03	1.2E-02
3-ketosphinganine	1.96	1.2E-03	1.2E-02
Sulfatide Ch18-0	0.55	1.3E-03	1.3E-02
SAM (S-adenosyl-L-methionine)	0.25	1.5E-03	1.4E-02
Sphingosine C18	1.97	1.6E-03	1.5E-02
Leukotriene A4	0.29	1.8E-03	1.6E-02
Sulfatide C26-1	1.44	2.4E-03	2.1E-02
Oleoylcarnitine	1.89	2.5E-03	2.2E-02
oxo-Resolvin D1	2.61	2.9E-03	2.4E-02
TXB2, 11-dehydro-2,3-dinor-	0.26	3.3E-03	2.7E-02
O-D-galactopyranosyl-N-hexacosanoylsphinganine	0.39	3.7E-03	2.9E-02
CerP d18:1,8:0	1.50	3.9E-03	3.1E-02
Sphingosine C17	2.27	6.2E-03	4.7E-02
L-Homotyrosine	1.65	6.5E-03	4.8E-02
Leukotriene B4, omega-carboxy-trinor-	0.37	6.8E-03	4.9E-02
5-Ketopalmitic acid	1.44	0.010	0.072
Hydroxymyristic acid	1.57	0.011	0.072
PIP 16-0,18-0	0.37	0.011	0.072
Sulfatide Ch16-0	0.62	0.014	0.093
D-Alanyl-D-Ser	2.60	0.018	0.111
Palmitoleic acid	1.57	0.018	0.111
Stearamide	1.50	0.021	0.125
PI 18-1,18-1	0.68	0.022	0.130
N-Arachidonoylsphinganine	0.52	0.023	0.130
Oleylarachidonate	2.11	0.028	0.154
Palmitoylglycerophosphocholine	1.47	0.034	0.182
Ubiquinone-4 (Coenzyme Q4)	1.47	0.035	0.182
Cholesterol	1.36	0.038	0.195
Sphinganine C18	1.73	0.042	0.213
LPC 16-0	1.47	0.048	0.222
PGE2 (prostaglandin)	0.60	0.049	0.222
GA2 d18-1,16-0 (ganglioside)	1.50	0.049	0.222
LacCer d18-1,18-0	1.36	0.049	0.222

Table S3. ER sphingolipids analysis for U251 ^{WT}, U251^{R132H} and U251^{R132H}+ AGI5198.

ID	*f*.value	*p*.value	FDR	Tukey's post-hoc ANOVA tests
CerP d18-1, 2:0 (N-acetyl-S1P)	415.4	3.69E-07	1.18E-05	ER_RH-ER_1WT; ER_RHA-ER_1WT; ER_RHA-ER_RH
Sphingosine d18-1	124.6	1.30E-05	1.55E-04	ER_RH-ER_1WT; ER_RHA-ER_1WT; ER_RHA-ER_RH
SM d18-2,24-1	120.0	1.45E-05	1.55E-04	ER_RH-ER_1WT; ER_RHA-ER_1WT; ER_RHA-ER_RH
Cer d18-1, 16-1	84.3	4.07E-05	2.68E-04	ER_RH-ER_1WT; ER_RHA-ER_RH
Cer d18-1,24-2	83.4	4.19E-05	2.68E-04	ER_RH-ER_1WT; ER_RHA-ER_RH
Cer d16-1,22-1	71.9	6.42E-05	2.97E-04	ER_RH-ER_1WT; ER_RHA-ER_1WT; ER_RHA-ER_RH
Cer d18-1,20-0	69.2	7.16E-05	2.97E-04	ER_RH-ER_1WT; ER_RHA-ER_1WT; ER_RHA-ER_RH
GlcCer d14-1,24-1-2OH	68.3	7.43E-05	2.97E-04	ER_RH-ER_1WT; ER_RHA-ER_1WT
GlcCer d18-1,24-1	65.6	8.35E-05	2.97E-04	ER_RH-ER_1WT; ER_RHA-ER_RH
GlcCer d16-2,24-0-2OH	47.3	2.12E-04	6.73E-04	ER_RH-ER_1WT; ER_RHA-ER_1WT
GlcCer d18-1,24-1-2OH	44.6	2.50E-04	6.73E-04	ER_RH-ER_1WT; ER_RHA-ER_1WT; ER_RHA-ER_RH
dhCer d18-0,22-0	44.5	2.53E-04	6.73E-04	ER_RH-ER_1WT; ER_RHA-ER_1WT; ER_RHA-ER_RH
GlcCer d18-1,22-0	42.1	2.95E-04	6.75E-04	ER_RH-ER_1WT; ER_RHA-ER_1WT; ER_RHA-ER_RH
GlcCer d18-1,24-0	42.0	2.95E-04	6.75E-04	ER_RH-ER_1WT; ER_RHA-ER_RH
GlcCer d18-1,22-0-2OH	27.8	9.29E-04	1.98E-03	ER_RH-ER_1WT; ER_RHA-ER_1WT

N-palmitoylserine	25.8	1.13E-03	2.27E-03	ER_RH-ER_1WT; ER_RHA-ER_RH
GlcCer d18-1,23-0	24.5	1.29E-03	2.32E-03	ER_RH-ER_1WT; ER_RHA-ER_RH
LacCer d18-0,18-0	24.4	1.31E-03	2.32E-03	ER_RH-ER_1WT; ER_RHA-ER_RH
GlcCer d18-1,23-1	21.1	1.93E-03	3.26E-03	ER_RHA-ER_1WT; ER_RHA-ER_RH
Lc3Cer d18-1,18-0 (Ganglioside)	20.0	2.21E-03	3.54E-03	ER_RH-ER_1WT; ER_RHA-ER_RH
Cer d18-1,24-1	18.6	2.66E-03	4.06E-03	ER_RH-ER_1WT; ER_RHA-ER_RH
GlcCer d18-1,24-0-2OH	17.4	3.20E-03	4.66E-03	ER_RH-ER_1WT; ER_RHA-ER_1WT
GlcCer d18-1,22-1	15.1	4.58E-03	6.37E-03	ER_RHA-ER_1WT; ER_RHA-ER_RH
GlcCer d18-1,18-0	13.6	5.95E-03	7.93E-03	ER_RH-ER_1WT; ER_RHA-ER_1WT
Sphinganine d18-0	7.7	2.21E-02	2.81E-02	ER_RHA-ER_1WT
Cer d18-0,18-1	7.6	2.28E-02	2.81E-02	ER_RHA-ER_RH
Cer d18-1,18-1	4.7	5.84E-02	6.88E-02	ER_RH - ER_1WT; ER_RH - ER_RHA
Sulfatide d18-1,24-1	3.5	9.90E-02	1.13E-01	ER_1WT - ER_RH; ER_1WT - ER_RHA
Hydroxypalmitate	1.7	2.57E-01	2.83E-01	ER_1WT - ER_RH; ER_1WT - ER_RHA; ER_RHA - ER_RH
Sulfatide d18-1,18-0-2OH	1.5	2.99E-01	3.19E-01	ER_1WT - ER_RH; ER_RHA - ER_RH
L-Serine	1.3	3.42E-01	3.53E-01	ER_1WT - ER_RH; ER_RHA - ER_RH

Table S4. Histological and Methylation Types of Tissue Present in Clusters EC1-EC6.

**Original Subtype**	**EC1**	**EC2**	**EC3**	**EC4**	**EC5**	**EC6**
Classical	2.91	0.00	0.00	5.19	17.50	8.25
G-CIMP	0.58	0.00	0.58	0.00	2.50	3.09
IDHmut-codeletion	1.74	0.00	76.88	23.38	6.67	7.22
IDHmut-non-codeletion	75.58	39.13	16.76	44.16	5.83	41.24
IDHwt	13.95	56.52	2.31	19.48	16.67	19.59
Mesenchymal	2.33	4.35	0.58	3.90	26.67	8.25
Neural	1.16	0.00	0.00	0.00	11.67	10.31
Proneural	1.74	0.00	2.89	3.90	12.50	2.06
**Methylation Subtype**	**EC1**	**EC2**	**EC3**	**EC4**	**EC5**	**EC6**
Classic-like	7.06	17.39	2.33	7.79	34.65	9.09
Codeletion	1.76	0.00	78.49	25.97	7.92	7.95
G-CIMP-high	75.88	39.13	13.95	33.77	5.94	47.73
G-CIMP-low	1.76	0.00	2.33	7.79	2.97	1.14
LGm6-GBM	1.18	0.00	0.00	3.90	3.96	3.41
Mesenchymal-like	9.41	13.04	1.74	12.99	41.58	27.27
PA-like	2.94	30.43	1.16	7.79	2.97	3.41
Classic-like	7.06	17.39	2.33	7.79	34.65	9.09
**Gender**	**EC1**	**EC2**	**EC3**	**EC4**	**EC5**	**EC6**
female	49.04	54.55	47.65	38.89	31.03	33.7
male	50.96	45.45	52.35	61.11	68.97	66.3

Table S5. EC50 for SphC17, NDMS and combination treatments.

Cell Line (Type)	$EC_{50, Sph C17}$ (mM)	$EC_{50, NDMS}$ (mM)	$EC_{50, NDMS+ SphC17}$ (mM)
**GSC 923 (GBM IV, IDH1^{WT})**	N.D.	N.D.	N.D.
**BT142 (Astro III, IDH1^{mut})**	3.71 ± 0.8	3.60 ± 0.80	3.39 ± 0.90
**NCH612 (Oligo III, IDH1^{mut})**	11.06 ± 5.17	1.18 ± 0.45	0.51 ± 0.10
**TS603 (Oligo III, IDH1^{mut})**	N.D.	42.35 ± 16.42	5.02 ± 1.05

© 2020 by the authors. Licensee MDPI, Basel, Switzerland. This article is an open access article distributed under the terms and conditions of the Creative Commons Attribution (CC BY) license (http://creativecommons.org/licenses/by/4.0/).
